# Baseline trachoma prevalence in Guinea: Results of national trachoma mapping in 31 health districts

**DOI:** 10.1371/journal.pntd.0006585

**Published:** 2018-06-11

**Authors:** André Géopogui, Christelly Flore Badila, Mamadou Siradiou Baldé, Cece Nieba, Lamine Lamah, Steven D. Reid, Mohamed Lamine Yattara, Jean Jacques Tougoue, Jeremiah Ngondi, Ibrahim Foungotin Bamba, Joseph J. Amon, Anthony W. Solomon, Yaobi Zhang

**Affiliations:** 1 National Neglected Tropical Diseases Control Program, Ministry of Health, Conakry, Guinea; 2 Guinea Office, Helen Keller International, Conakry, Guinea; 3 Headquarters, Helen Keller International, New York, New York, United States of America; 4 Mali Office, Helen Keller International, Bamako, Mali; 5 ENVISION Project, RTI International, Washington DC, United States of America; 6 ENVISION Project, RTI International, Dar es Salaam, Tanzania; 7 Clinical Research Department, London School of Hygiene & Tropical Medicine, London, United Kingdom; 8 Department of Neglected Tropical Diseases, World Health Organization, Geneva, Switzerland; 9 Regional office for Africa, Helen Keller International, Dakar, Senegal; King Saud University College of Medicine, SAUDI ARABIA

## Abstract

**Background:**

Based on previous studies, historical records and risk factors, trachoma was suspected to be endemic in 31 health districts (HDs) in Guinea. To facilitate planning for the elimination of trachoma as a public health problem, national trachoma surveys were conducted between 2011 and 2016 to determine the prevalence of trachomatous inflammation—follicular (TF) and trachomatous trichiasis (TT) in all 31 endemic HDs.

**Methodology/Principal findings:**

A total of 27 cross-sectional surveys were conducted, each using two-stage cluster sampling (one survey in 2011 covered five HDs). Children aged 1–9 years and adults aged ≥15 years were examined for TF and TT, respectively, using the World Health Organization (WHO) simplified grading system. Indicators of household access to water, sanitation and hygiene (WASH) were also collected. A total of 100,051 people from 13,725 households of 556 clusters were examined, of whom 44,899 were male and 55,152 were female. 44,209 children aged 1–9-years and 48,745 adults aged ≥15 years were examined. The adjusted prevalence of TF varied between 1.0% (95%CI: 0.6–1.5%) to 41.8% (95%CI: 39.4–44.2%), while the adjusted prevalence of TT ranged from 0.0% (95%CI: 0.0–0.2%) to 2.8% (95%CI: 2.3–3.5%) in the 27 surveys. In all, 18 HDs had a TF prevalence ≥5% in children aged 1–9 years and 21 HDs had a TT prevalence ≥0.2% in adults aged ≥15 years. There were an estimated 32,737 (95% CI: 19,986–57,811) individuals with TT living in surveyed HDs at the time of surveys.

**Conclusions/Significance:**

Trachoma is a public health problem in Guinea. 18 HDs required intervention with at least one round of mass drug administration and an estimated 32,737 persons required TT surgery in the country. The results provided clear evidence for Guinea to plan for national trachoma elimination.

## Author summary

Trachoma is the leading infectious cause of blindness worldwide. The World Health Organization (WHO) recommends that endemic countries implement the SAFE strategy (surgery for trichiasis, antibiotic treatment, facial cleanliness and environmental improvement) to achieve trachoma elimination by the year 2020. Trachoma was suspected to be endemic in Guinea in 31 health districts except those in and around the capital Conakry, based on historical records and previous studies. To facilitate planning for the elimination of trachoma as a public health problem, Guinea conducted 27 separate trachoma surveys between 2011 and 2016 to determine the prevalence of trachomatous inflammation—follicular (TF) and trachomatous trichiasis (TT) in these 31 health districts. The results showed 18 health districts requiring intervention with at least one round of mass drug administration and an estimated 32,737 persons requiring TT surgery in the country. These data provided clear evidence for Guinea to plan for national trachoma elimination.

## Introduction

Trachoma, caused by ocular infection with *Chlamydia trachomatis* [1, 2], is the leading infectious cause of blindness worldwide. It affects those living in poverty, predominantly in rural areas, where access to water and sanitation is limited [3]. In 1998, the World Health Assembly adopted Resolution 51.11 which targets the global elimination of blinding trachoma [4]. The World Health Organization (WHO) endorses the “SAFE” strategy for trachoma elimination: **S**urgery for trachomatous trichiasis (TT), **A**ntibiotic treatment for infection, **F**acial cleanliness and **E**nvironmental improvement to reduce transmission [5–7]. Prior to the implementation of the SAFE strategy, disease mapping is a critical first stage to determine the distribution of the disease and determine whether public health interventions are needed. The provision of accurate estimates of the prevalence of both active trachoma, mainly trachomatous inflammation–follicular (TF), and TT enables national programs to plan and implement mass drug administration (MDA) and surgical services respectively [8].

Guinea is a country in West Africa with an estimated population of 12 million [9] and a total area of 245,857 km^2^. The country is bordered on the west by the Atlantic Ocean, on the north by Guinea-Bissau and Senegal, on the east by Mali and Côte d'Ivoire, and on the south by Liberia and Sierra Leone. Guinea has a tropical climate of alternating rainy season and dry season of approximately six months each. It has four natural regions, including Lower Guinea or Maritime Guinea, Middle Guinea, Upper Guinea and Forest Guinea. Lower Guinea is a coastal plain which covers 18% of the national territory with a climate characterized by heavy precipitation ranging from 3,000 to 4,000 mm per year, and high humidity. Middle Guinea is a mountainous area which covers 22% of the national territory, with annual rainfall of 1,500 to 2,000 mm and a semi temperate climate. Upper Guinea is a region of plateaus and savanna woodland which covers 40% of the country. Precipitation ranges from 1,000 to 1,500 mm per year, with a dry and hot climate. Finally, Forest Guinea is a group of massifs and covers 20% of the national territory, characterized by an annual rainfall ranging between 2,000 and 3,000 mm with a humid climate. The country is split into eight administrative regions and 38 health districts (HDs).

Trachoma was suspected to be endemic in Guinea in 31 rural HDs, but not in 7 HDs in and around the capital Conakry, according to historical clinical records and a limited number of epidemiological surveys [10–12]. In 2001 an epidemiological survey was conducted in 10 of the 31 suspected HDs: nine in Upper Guinea (Dabola, Dinguiraye, Faranah, Kankan, Kérouané, Kissidougou, Kouroussa, Mandiana and Siguiri) and one HD in the forest region (Beyla). The survey showed a prevalence of 33% of active trachoma (including TF and trachomatous inflammation–intense [TI]) among children under the age of 10 years, and 2.7% of TT among adults aged 15 and above [5, 10, 13]. In 2002, a trachoma rapid assessment [14] was undertaken in five other suspected endemic districts in Middle Guinea (Gaoual, Koubia, Koundara, Mali and Tougué) and showed an average TF and TT prevalence of 23.0% and 1.1% respectively [10]. These survey results showed that the country was indeed endemic for trachoma and warranted major intervention to achieve the objective of trachoma elimination by the year 2020.

In 2011, Guinea initiated an integrated national program to control and eliminate neglected tropical diseases (NTDs), including trachoma. In order to plan for trachoma elimination activities, up-to-date accurate district-level estimates of trachoma prevalence were needed. From 2011 to 2016, the national NTD program conducted trachoma baseline surveys. The results of these surveys are presented here along with discussion of their impact on intervention planning for trachoma elimination in Guinea.

## Materials and methods

### Survey settings and design

A series of cross-sectional, two-stage cluster sampling surveys were used to determine the prevalence of TF and TT in 31 HDs in Guinea, as recommended by WHO [8, 15, 16]. Surveys were conducted in multiple phases from 2011 to 2016, according to the schedule of the integrated national NTD program, and used to develop a national map of trachoma prevalence. The timeline for specific districts were as follows:

2011: Baseline surveys were conducted in five HDs in Upper Guinea (Dabola, Dinguiraye, Faranah, Kissidougou and Kouroussa). These five HDs were among the 10 HDs in the original 2001 epidemiological survey and had previously been shown to be highly endemic for trachoma [10]. The 2011 survey was conducted to confirm that prevalence was still high and to collect baseline data in these HDs before starting MDA. These five HDs were surveyed together as a single evaluation unit (EU).

2012–13: Two HDs in 2012 (Koundara in Middle Guinea and Yomou in Forest Guinea) and nine HDs in 2013 (Gaoual, Koubia, Mali and Tougué in Middle Guinea, Kankan, Kérouané, Mandiana and Siguiri in Upper Guinea and Beyla in Forest Guinea) were surveyed. These 11 HDs were surveyed as 11 separate EUs.

2014–16: 11 HDs in Lower Guinea (Boffa, Boké, Forécariah, Fria, Kindia and Télimélé) and Middle Guinea (Dalaba, Labe, Lélouma, Mamou and Pita) were surveyed in 2014. Due to the outbreak of Ebola virus disease, survey of the remaining four HDs in Forest Guinea (Guéckédou, Macenta, N’zérékoré and Lola) was delayed and completed between December 2015 and February 2016. These 15 HDs were surveyed as 15 distinct EUs, using the systems and processes of the Global Trachoma Mapping Project (GTMP) [17].

### Target population and sample size

For the 2011 survey, data on the total population of the villages belonging to the five targeted HDs were available from the community directed treatment with ivermectin (CDTI) report of 2010 [18]. From 2012 to 2014, population data were estimated from the 1996 national census [19], and for the 2016 survey, the population was projected from the 2014 national census [20].

The target population was children aged 1–9 years and adults aged ≥15 years for estimating the prevalence of TF and TT, respectively. Although these age groups were of primary importance of the surveys, all individuals over the age of 1 year living in selected households were screened for signs of trachoma, and data from all examined individuals were recorded. Any person who had resided in a sampled household for at least one month prior to the survey date was considered eligible for inclusion. People who refused to participate or were absent at the time of survey were not replaced.

To estimate the EU-level prevalence of TF, a sample size of children aged 1–9 years was calculated for each EU, based on an expected TF prevalence of 10% [16, 17, 21], with a 95% confidence level, an absolute precision of 3%, and a design effect (to adjust for cluster sampling) of 2.65. A minimum sample size of 1,019 children aged 1–9 years was needed. To allow for 20% non-response, we tried to enroll 1,273 children aged 1–9 years in each EU.

We did not calculate a sample size for TT prevalence in adults aged ≥15 years; instead, having determined the number of households required to recruit sufficient children to estimate TF prevalence, all consenting adults aged ≥15 years living in the same households were examined to estimate the TT prevalence in each EU.

### Selection of clusters and households

The sampling frames comprised all the villages in each EU and their respective populations. The protocol followed WHO recommendations [16], with 20 villages and 30 households per village chosen from each EU. However, in certain EUs where there was a low average number of 1–9-year-old children per household, the number of villages to be surveyed in each EU was increased. In the first sampling stage, a list of all the villages was obtained for each EU, and 20 villages were selected systematically through a probability proportional to population size sampling strategy [16]. In the 2011 survey, which covered five HDs as a single EU, a total of 30 villages were purposively selected, with six drawn from each HD. In all cases, only villages with populations ranging from 300 to 5,000 inhabitants were eligible for selection. In the second sampling stage, all households from each selected village were listed, and 30 were randomly selected through the compact segment sampling method [14, 21].

Within selected households, all consenting/assenting individuals over the age of one year were examined for trachoma signs. Heads of households were interviewed on key water, sanitation and hygiene (WASH) indicators using paper forms for 2011–2013 surveys and a standard Android phone-based questionnaire [22] developed by GTMP for the 2014–16 surveys.

### Training

Graders were recruited from the existing pool of Ophthalmic Clinical Officers (OCOs) in Guinea. Training was provided to graders on examination of community residents in the sampled households for clinical signs of trachoma. The training of graders in 2014 and 2016 was performed by GTMP-certified grader-trainers. For the recognition of signs of trachoma, slides showing pictures of various forms of trachoma were used in classroom sessions. Classroom training was followed by a field test and certification. All the graders participating in the surveys had obtained a kappa for diagnosing TF of at least 0.7 in a formal inter-grader agreement test (based on a sample of 50 children), compared to a GTMP certified grader trainer.

Recorders were also trained to operate the Android devices and enter trachoma grading data from each eligible person as well as WASH data from each household visited. The recruitment of recorders was based on their knowledge and skills to operate and manage electronic devices at ease.

### Household survey

Each survey team was comprised of one grader, one recorder and one community member from the sampled village (acting as a guide/translator). All the teams conducted surveys district by district and household by household, and they were supervised by one supervisor.

All residents in the household were enumerated, including registration of details of their age and gender. Household members aged one year and above, present at the time of survey and willing to participate in the surveys were examined by a grader with a 2.5x magnifying loupe and a torch or sunlight. The WHO simplified trachoma grading system was used [23, 24].

Graders used gloves and alcohol-based hand gel to clean their hands before examining each participant to avoid spreading any potential infections, including Ebola. Prior to the eye examination, facial cleanliness for children aged 1–9 years was observed. Facial cleanliness was defined as the absence of nasal and ocular discharge. Basic treatment using drugs such as 1% tetracycline eye ointment and analgesics were provided in the field for those in need. Patients needing referral to a hospital were referred promptly. Field teams moved from one selected household to the next until all the 30 selected households in the village were surveyed.

### Data collection, management and analysis

In the 2011–2013 surveys, data were collected and recorded on paper forms. The data included individual trachoma grading of both eyes of each subject, and household data, such as information on latrine and water sources. The data from 2011 survey were entered into Epi Info software (CDC, Atlanta, US) and those from 2012 and 2013 were entered into CS-PRO software (Census Bureau and ICF Macro, US).

From 2014 to 2016, the GTMP data collection tools were used. Briefly, these were electronic data capture forms running on Android smartphones within the GTMP-LINKS application (Task Force Links/Task Force for Global Health, Decatur, GA, USA) [17, 25]. Data collected in the field were transferred securely from the field to a central cloud-based reporting and data management system.

Data from all surveys were transferred into SPSS (IBM, version 23) for analysis. Descriptive statistics were used to examine the sample characteristics, the prevalence of trachoma, and the proportion of households with key WASH indicators. The adjusted prevalence of TF and TT was estimated according to the methods described previously [17], i.e. adjusting the prevalence according to age and gender using the 2014 Guinean national population as the standard population [26], except that the adjustment was made at the EU level rather than the village level. For TF prevalence in each EU, the proportion of 1–9-year-olds examined was adjusted by weighting the proportion of each 1-year age band examined by the proportion of that age band in the national 1–9-year-old population. For TT prevalence in each EU, the proportion of ≥15-year-olds examined was adjusted by weighting the proportion of each gender-specific 5-year age band examined by the proportion of that gender-specific age band in the national ≥15-year-old population. The 95% confidence intervals (CIs) of prevalence estimates were calculated using the Wilson score method without continuity correction [27].

### Ethical considerations

The surveys were part of the routine disease surveillance activities of the national trachoma elimination program of the Ministry of Health (MoH), Guinea. It was a standard public health measure and all procedures followed WHO recommendations. Protocols were approved by the national Ethics Committee of MoH, and, for the 2014–2016 surveys, by the research ethics committee of the London School of Hygiene & Tropical Medicine (6319). Prior to examination, verbal informed consent or assent was obtained from all adult participants and for children from the head of the household. Those with active trachoma were provided with 1% tetracycline eye ointment.

## Results

### Survey population characteristics by district

Table 1 summarizes the characteristics of the survey populations by EU. In total, 13,725 households were visited in 556 villages of 31 HDs. A total of 102,040 people were enumerated: 46,048 males and 55,992 females in 31 districts. Among these, a total of 100,051 people were examined (44,899 males and 55,152 females), representing a participation rate of 98.1% (97.5% for males and 98.5% for females) (Table 1). The number of children aged 1–9 years examined was 44,209 and the number of the adults aged ≥15 years examined was 48,745.

**Table 1:** Characteristics of surveyed population by evaluation unit in Guinea.

District	Year of survey	Estimated population during the baseline survey^a^	Number of villages surveyed	No of households selected	No of residents enumerated	Total examined	No of children 1–9 years old examined	No of persons ≥15 years old examined
Dabola, Dinguiraye, Faranah, Kissidougou, Kouroussa^b^	2011	1,096,933	30	655	2,416	2,416	1,617	799
Koundara	2012	119,984	20	483	4,687	4,537	2,021	2,069
Yomou	2012	358,413	20	469	5,547	5,271	1,995	2,623
Beyla	2013	263,944	20	400	4,402	4,294	1,904	2,323
Gaoual	2013	193,263	20	502	4,567	4,543	1,925	2,276
Kankan	2013	425,476	20	436	4,832	4,591	2,178	2,294
Kérouané	2013	298,436	21	429	4,803	4,731	2,016	2,318
Koubia	2013	125,780	20	418	4,277	4,228	1,739	2,256
Mali	2013	232,860	20	447	4,718	4,697	2,338	2,281
Mandiana	2013	287,203	23	461	5,417	5,292	2,329	2,626
Siguiri	2013	491,506	22	442	5,468	5,303	2,429	2,621
Tougué	2013	174,058	20	427	4,908	4,823	2,057	2,380
Boffa	2014	234,624	20	467	3,260	3,250	1,539	1,449
Boké	2014	569,271	20	506	3,110	3,101	1,375	1,476
Dalaba	2014	211,670	20	563	2,667	2,507	909	1,332
Forécariah	2014	481,466	20	497	2,917	2,906	1,266	1,373
Fria	2014	139,451	20	480	3,173	3,159	1,505	1,402
Kindia	2014	569,732	20	539	3,282	3,275	1,515	1,468
Labé	2014	372,152	20	539	2,791	2,648	988	1,339
Lélouma	2014	167,257	20	524	2,890	2,853	1,190	1,438
Mamou	2014	444,198	20	535	2,954	2,915	1,312	1,357
Pita	2014	307,203	20	558	2,815	2,763	1,124	1,423
Télimélé	2014	304,012	20	551	2,858	2,819	1,207	1,321
N’Zérékoré	2015	414,607	20	597	3,531	3,490	1,460	1,800
Guéckédou	2016	303,539	20	603	3,259	3,194	1,345	1,618
Lola	2016	179,193	20	599	3,033	3,012	1,344	1,479
Macenta	2016	290,843	20	598	3,458	3,433	1,582	1,604
**Total**	** **	**9,057,074**	**556**	**13,725**	**102,040**	**100,051**	**44,209**	**48,745**

Note

^a^ 2011–2013: projection based on 1996 census, 2014–2016: based on 2014 census.

^b^ These HDs were surveyed together as one EU with 6 villages per HD.

### Prevalence of TF in children aged 1–9 years

**Fig 1:**
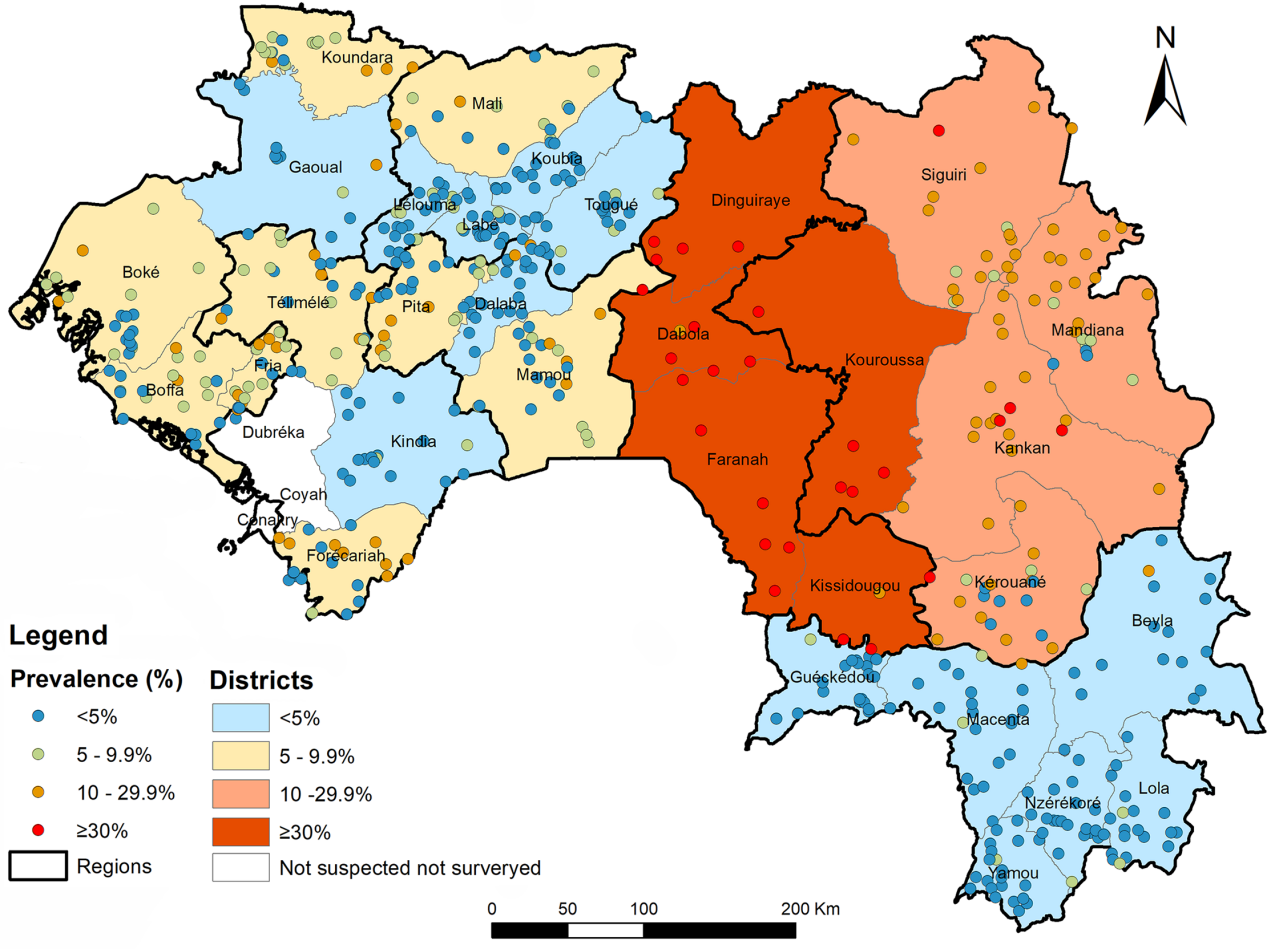
Health district TF endemicity categories and cluster-level distribution in Guinea. GPS coordinate information was missing for 24 clusters from the 2011–13 surveys and these clusters are not shown in the map. Figure was created for this publication using shapefiles from the GADM database (www.gadm.org), version 2.8, November 2015 and ArcGIS 10.4.1 for Desktop (ESRI, Redlands, CA).

The adjusted prevalence of TF in children aged 1–9 years is shown for each EU in Table 2. There was a wide variation in the TF prevalence among EUs, ranging from 1.0% in Yomou district to 41.8% in Dabola, Dinguiraye, Faranah, Kissidougou and Kouroussa EU. The HD-level TF prevalence categories and the cluster TF prevalence distribution are shown in Fig 1. Fourteen EUs (encompassing eighteen HDs) showed a TF prevalence above the MDA intervention threshold of 5%. Among these, nine (9) HDs had a TF prevalence between 5% and 9.9% (Boffa, Boké, Forécariah, Fria, Koundara, Mali, Mamou, Pita and Telimélé in Middle and Lower Guinea). Four (4) HDs had a TF prevalence between 10% and 29.9% (Kankan, Kérouané, Mandiana and Siguiri in Upper Guinea). Five (5) HDs had a TF prevalence of 30% or more (Dabola, Dinguiraye, Faranah, Kissidougou and Kouroussa, all in Upper Guinea). Thirteen of the 31 HDs surveyed had a TF prevalence of less than 5%, including all the HDs of Forest Guinea.

**Table 2:** Adjusted prevalence of TF, TT and estimated TT cases in Guinea.

District	Year of survey	No of children (1–9 years) examined	No of persons (≥15 years) examined	Adjusted prevalence (%) of TF (95% CI) in children 1–9 years	Adjusted prevalence (%) of TT (95% CI) in population (≥15 years)	Estimated TT backlog (95% CI) in population (≥15 years)^a^
Dabola, Dinguiraye, Faranah, Kissidougou, Kouroussa^b^	2011	1,617	799	41.79 (39.41–44.21)	2.02 (1.25–3.25)	12,242 (7,572–19,697)^c^
Koundara	2012	2,021	2,069	9.2 (8.0–10.59)	0.6 (0.4–1.1)	407 (237–699)
Yomou	2012	1,995	2,623	1.0 (0.6–1.5)	0.1 (0.0–0.2)	101 (0–470)
Beyla	2013	1,904	2,323	2.4 (1.8–3.1)	0.3 (0.1–0.6)	414 (197–872)
Gaoual	2013	1,925	2,276	3.6 (2.8–4.5)	0.6 (0.3–1.0)	620 (364–1,054)
Kankan	2013	2,178	2,294	25.8 (24.0–27.7)	1.2 (0.8–1.7)	2,840 (1,964–4,100)
Kérouané	2013	2,016	2,318	10.3 (9.1–11.7)	0.2 (0.1–0.5)	366 (158–845)
Koubia	2013	1,739	2,256	2.2 (1.6–3.1)	0.1 (0.0–0.4)	88 (0–265)
Mali	2013	2,338	2,281	5.8 (4.9–6.8)	0.5 (0.3–0.9)	620 (346–1,107)
Mandiana	2013	2,329	2,626	16.6 (15.1–18.1)	0.5 (0.3–0.9)	842 (501–1,412)
Siguiri	2013	2,429	2,621	16.9 (15.5–18.5)	2.8 (2.3–3.5)	7,700 (6,152–9,623)
Tougué	2013	2,057	2,380	3.3 (2.6–4.1)	0.1 (0.0–0.3)	45 (0–237)
Boffa	2014	1,539	1,449	6.2 (5.1–7.5)	0.1 (0.0–0.4)	89 (0–505)
Boké	2014	1,375	1,476	6.0 (4.9–7.4)	0.4 (0.2–0.9)	1,358 (637–2,890)
Dalaba	2014	909	1,332	2.0 (1.2–3.1)	0.2 (0.1–0.6)	190 (55–662)
Forécariah	2014	1,266	1,373	7.4 (6.1–8.9)	0.2 (0.1–0.7)	636 (226–1,786)
Fria	2014	1,505	1,402	8.5 (7.2–10.1)	0.1 (0.0–0.5)	90 (0–368)
Kindia	2014	1,515	1,468	2.6 (1.9–3.5)	0.0 (0.0–0.3)	71 (0–958)
Labé	2014	988	1,339	2.7 (1.8–3.9)	0.8 (0.4–1.4)	1,632 (904–2,941)
Lélouma	2014	1,190	1,438	3.4 (2.5–4.5)	0.2 (0.1–0.6)	163 (0–521)
Mamou	2014	1,312	1,357	5.8 (4.7–7.2)	0.3 (0.1–0.7)	671 (253–1,780)
Pita	2014	1,124	1,423	7.1 (5.7–8.7)	0.3 (0.1–0.7)	467 (180–1,209)
Télimélé	2014	1,207	1,321	7.0 (5.7–8.6)	0.4 (0.1–0.8)	585 (241–1,412)
N’Zérékoré	2015	1,460	1,800	1.4 (0.9–2.2)	0.1 (0.0–0.4)	231 (0–889)
Guéckédou	2016	1,345	1,618	1.7 (1.1–2.6)	0 (0–0.2)	0 (0–398)
Lola	2016	1,344	1,479	1.4 (0.9–2.2)	0.1 (0.0–0.5)	124 (0–472)
Macenta	2016	1,582	1,604	1.4 (0.9–2.1)	0.1 (0.0–0.4)	144 (0–640)
**TOTAL**	** **	**44,209**	**48,745**			**32,737 (19,986–57,811)**

Note

^a^ TT backlog was estimated according to the population of ≥15 years which accounts for 55.3% of the total population in Guinea [26].

^b^ These HDs were surveyed together as one EU. ^c^ According to the proportion of population in the five HDs, the estimated TT backlog was 1,937 in Dabola, 2,178 in Dinguiraye, 2,277 in Faranah, 3,449 in Kissidougou, and 2,400 in Kouroussa.

### Prevalence of TT and estimation of TT cases in adult population (15 years and older)

**Fig 2:**
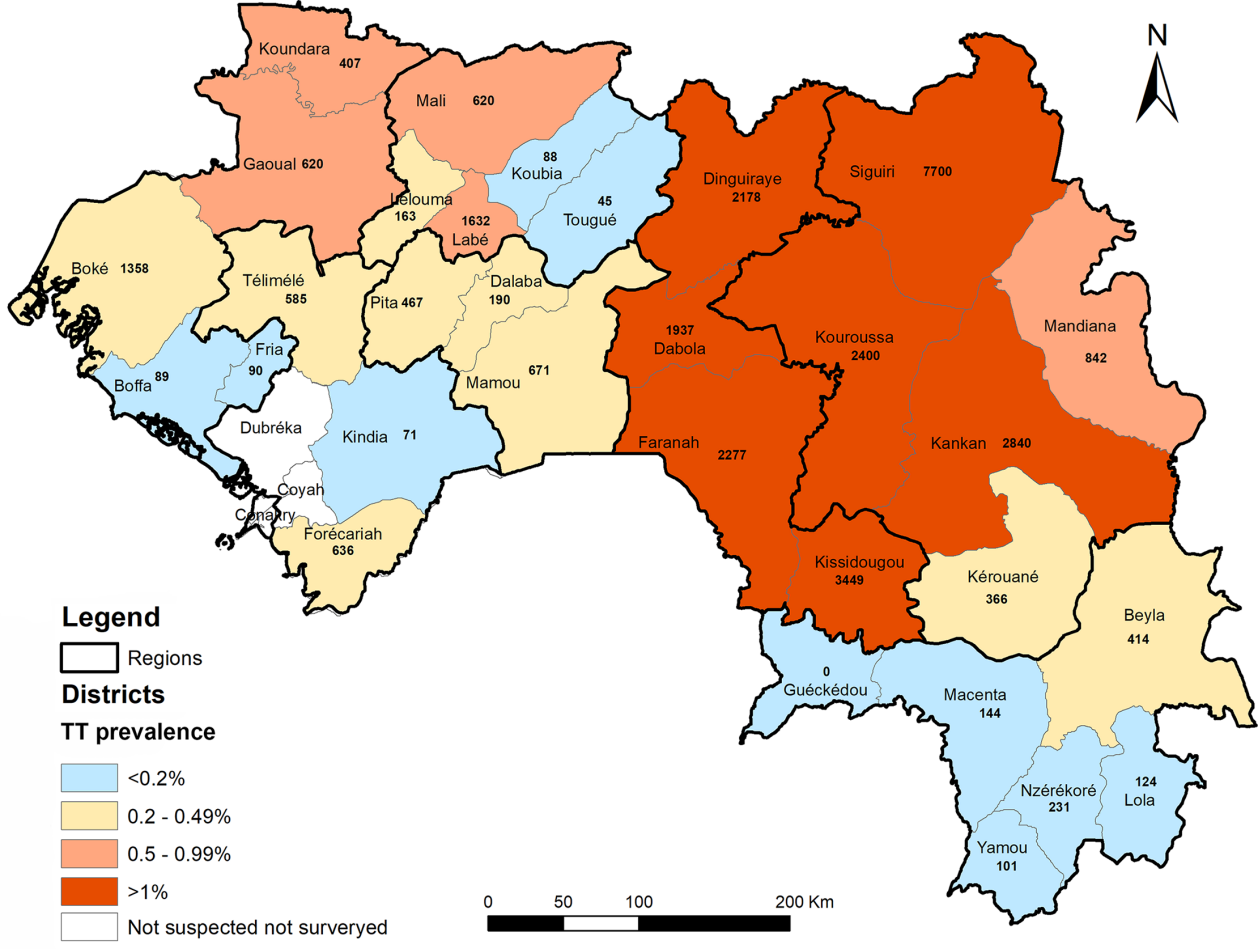
Health district TT prevalence categories and cases in Guinea. Numeric numbers represent the estimated number of TT cases in population aged ≥15 years in each HD. Figure was created for this publication using shapefiles from the GADM database (www.gadm.org), version 2.8, November 2015 and ArcGIS 10.4.1 for Desktop (ESRI, Redlands, CA).

As shown in Table 2, among 27 EUs (31 HDs), estimated prevalence of TT ranged from 0% (95% CI: 0–0.2%) in Guéckédou HD to 2.8% (95% CI: 2.3–3.5%) in Siguiri HD. Twenty-one (21) HDs had prevalence of TT of ≥ 0.2% (Table 2 and Fig 2). The total TT cases in the 31 HDs are estimated at 32,737 (95% CI: 19,986–57,811 cases). Dabola, Dinguiraye, Faranah, Kankan, Kissidougou, Kouroussa and Siguiri HDs had most TT cases.

### Household access to water, sanitation and hygiene

The key WASH indicators by EU are shown in Table 3. Between 58% and 100% of households in the surveyed EUs reported drinking water source located less than 30 minutes or within 1 km walking distance. Tougué HD had the lowest proportion (58%) of households with a drinking water source located less than 30 minutes. An average of 67% households had improved sanitation facilities (ranging between 17.1% in Mali HD and 96% in Kankan HD). In the 2013 survey, more than 52% of toilets were poorly maintained (detail not shown). Also in the 2013 survey, the TF prevalence was higher in the population living in households with poorly maintained toilets (38.9%), compared with 24.5% for households with well-maintained toilets (χ^2^ = 65.404, p<0.001).

**Table 3:** Key indicators on access to water, sanitation and hygiene (WASH) by district in Guinea.

District	Year of survey	No. of households surveyed	% of households with drinking water less than 1 km (30 min)	% of households having improved sanitation facilities
Dabola, Dinguiraye, Faranah, Kissidougou, Kouroussa^a^	2011	655	99.5	62
Koundara	2012	483	100	53.2
Yomou	2012	469	100	33.9
Beyla	2013	400	69	64.1
Gaoual	2013	502	86.3	62.5
Kankan	2013	436	93.2	95.7
Kérouané	2013	429	84.2	90
Koubia	2013	418	70.1	49.9
Mali	2013	447	63.3	17.1
Mandiana	2013	461	70.4	86.4
Siguiri	2013	442	84.8	91.2
Tougué	2013	427	57.7	70.8
Boffa	2014	467	87	76.4
Boké	2014	506	78.1	84.3
Dalaba	2014	563	88.3	78.1
Forécariah	2014	497	69.1	83.2
Fria	2014	480	81	54
Kindia	2014	539	82.9	81.3
Labé	2014	539	82.7	85.6
Lélouma	2014	524	82.2	65.7
Mamou	2014	535	77.1	67.3
Pita	2014	558	71	75.8
Télimélé	2014	551	68.6	50
N'Zérékoré	2015	597	90.4	67.8
Guéckédou	2016	603	80.6	46.8
Lola	2016	599	98.6	44.2
Macenta	2016	598	98	82.5
**Average**	** **	** **	**82.7**	**67.2**

Note

^a^ These HDs were surveyed together as one EU.

## Discussion

The results of the national trachoma mapping revealed that trachoma was indeed a public health problem in Guinea. Among the 31 HDs suspected to be endemic for trachoma, 18 HDs had TF prevalence estimates above the WHO threshold of ≥5% and required district-level intervention with antibiotic MDA, as well as implementation of the, F and E components of SAFE. The total population in need of at least one round of MDA in these 18 HDs was approximately 5.5 million. Among the 18 HDs, nine HDs (Boffa, Boké, Forécariah, Fria, Koundara, Mali, Mamou, Pita and Télimélé) had TF prevalence estimates between 5% and 9.9% and therefore required implementation of one round of MDA before undertaking impact surveys. Four HDs (Kankan, Kérouané, Mandiana and Siguiri) had a TF prevalence of 10% to ≤29.9% requiring implementation of at least three rounds of MDA before undertaking impact surveys. Five districts (Dabola, Dinguiraye, Faranah, Kissidougou and Kouroussa) had TF prevalence estimates of ≥30% and therefore needed implementation of at least 5 rounds of MDA before impact surveys. The remaining 13 HDs had TF prevalence of below 5% thus implementation of antibiotic MDA is not required.

The adjusted prevalence of TT in the 31 surveyed HDs varied from 0.0% to 2.8%. Twenty-one HDs had TT prevalence of ≥0.2%. There were an estimated 32,737 persons requiring TT surgical intervention. To eliminate trachoma as a public health problem, the health system has to ensure that the prevalence of TT unknown to the health system is reduced to less than 2 in 1000 people aged ≥15 years. In the 31 HDs, this means that the minimum number of TT patients that should be offered surgery to reach the elimination threshold, ignoring incident cases and mortality in those with TT, was 21,507 at the time of survey [8].

In general, HDs in Upper Guinea had the highest trachoma prevalence and the most TT cases requiring surgery in the country (Figs 1 and 2). The five HDs (Dabola, Dinguiraye, Faranah, Kissidougou and Kouroussa,) that showed high prevalence in the 2001 survey [13, 28], had this impression confirmed in the 2011 survey results. This part of Guinea borders with the Kayes, Koulikoro and Sikasso regions of Mali where high prevalence of trachoma has also been noted [29, 30]. The HDs in Middle and Lower Guinea showed low trachoma prevalence, though there were some clusters in which more than 10% of examined children had TF (Fig 1). The presence here of some village-level TF proportions exceeding 10% should not cause alarm: an exponential distribution of cluster-level proportions is predicted to be present when an infectious disease is disappearing–and such a distribution has a tail with a few high-burden clusters [31]. None of the HDs in the Forest Guinea require antibiotic MDA. This is also consistent with the situation in Guinea’s neighboring countries, such as Cote d’Ivoire, Guinea Bissau, Liberia, Senegal and Sierra Leone, [13, 32, 33]. Overall, Upper Guinea is characterized by a dry season, lowest precipitation, and high year-round temperatures. Climatic and environmental factors may have influenced the distribution and prevalence of trachoma in Guinea via a number of mechanisms, including via influence on the distribution, abundance or seasonal activity of *Musca sorbens*, the principal eye-seeking fly implicated in trachoma transmission [30, 34–37].

These mapping results provided evidence that has informed Guinea’s national plan to eliminate trachoma as a public health problem by 2020. Antibiotic MDA started in 2013 in two districts and has since been scaled up to cover, by 2017, all 18 HDs that require it. In addition to the implementation of MDA, these 18 HDs also required the implementation of the F and E components of SAFE for trachoma elimination. The lack of availability of toilets/latrines for the safe disposal of human faeces was found in Koubia, Koundara, Kissidougou and Mali HDs (50%, 46%, 33% and 17% respectively). It was noted that in all the 31 evaluated HDs, most water sources were within 1 km of the household. There was not, in the survey results, a clear link between the availability of toilets or the presence of drinking water less than 30 minutes and trachoma prevalence. A recent paper analyzed multi-country trachoma survey data which included 2014–2016 survey data from Guinea, found no apparent threshold between community-level water coverage and TF prevalence [38]. However, it was observed in the 2013 survey that more than half of all latrines were poorly maintained, and potentially, therefore, acting as *M*. *sorbens* breeding sites, or at the very least discouraging people from using them for their intended purpose.

There were a number of limitations to our surveys. Firstly, although the same sampling methodology was used in all surveys, the indicators collected in different phases of surveys were somewhat inconsistent. Analyses of associations of trachoma with risk factors using data from across the country were therefore not performed. Instead, we have emphasized generation of WHO-recommended indicators for trachoma elimination, to guide national program decision-making. Secondly, the 2011 survey of five highly-endemic HDs was undertaken to confirm the results collected 10 years before, in order to ensure that antibiotic MDA intervention was still justified. These five HDs were not surveyed as individual EUs, but as one EU with six clusters per HD. The villages sampled in this 2011 survey were selected from known highly-endemic villages based on previous survey data, and therefore may have overestimated the true trachoma prevalence for these HDs. We have not reported HD-level estimates for these HDs, around which confidence intervals would be expected to be very wide. We note that no interventions against trachoma had taken place in these HDs between the 2001 and 2011 surveys, and that the EU-level prevalence estimate therefore matched our pre-survey expectations, justifying interventions. Thirdly, the surveys took place over several years. Projected populations in the survey years were used to estimate the number of prevalent TT cases. This may have caused inaccurate estimation of TT backlogs for program purposes. More work is currently being done to refine the ways that programs estimate TT prevalence.

In conclusion, these surveys confirmed that trachoma was a public health problem in Guinea, with 18 HDs requiring intervention with at least one round of MDA and an estimated 32,737 people with TT requiring surgery. The data provide the evidence base for the Ministry of Health to plan for implementing the full WHO-endorsed SAFE strategy to eliminate trachoma as a public health problem from Guinea. By 2017, Guinea achieved complete national antibiotic coverage of areas that require it for trachoma elimination purposes, and impact surveys are now being conducted to assess whether the country is on track to achieve the year 2020 goal.

## Supporting information

S1 Checklist STROBE checklist. (DOC)
